# Is a Conservative Approach Justified in Penetrating Liver Injury?

**DOI:** 10.1155/1995/67150

**Published:** 1995

**Authors:** H. Leon Pachter

**Affiliations:** New York University School of Medicine Trauma-Shock Unit Bellevue Hospital NewYork City N.Y. USA


research should be directed at studies of the interplay between bowel integrity, portal circulation and liver function. Different preventative measures to increase the gut integrity in the form of selective antibiotic treatment, administration, of immunostimulants, stimulation of mucosal growth or induction of bacterial antagonism by the administration of probiotics require further evaluation.

With this in mind the answer to the question posed in the paper could be within the range of 60-90 minutes but the question should be extended: where is the limit for combined liver ischemia and splanchnic stasis?

*References*

1. Taniguchi, H., Takahashi, T., Shioaki, Y., Itoh, A., Oguro, A. (1992) Vascular inflow exclusion and hepatic resection. *Br. J. Surg.*, **79**, 672–675.
2. Hannoun, L., Borie, D., Delva, E., Jones, D., Vaillant, J-C., Nordlinger, B., Parc, R. (1993) Liver resection with normothermic ischemia exceeding 1 h. *Br. J. Surg.*, **80**, 1161–1165.
3. Kim, Y. I., Nakashima, K., Tada, I., Kawano, K., Kobayashi, M. (1993) Prolonged normothermic ischemia of human cirrotic liver during hepatectomy: a preliminary report. *Br. J. Surg.*, **80** 1566–1570.
4. Ekberg, H., Tranberg, K-G., Andersson, R., Jeppsson, B., Bengmark. S. (1986) Major liver resection-perioperative course and management. *Surgery*, **100**, 1–8.
5. Liu, L., Jeppsson, B., Bengmark, S. (1992) Bacterial translocation into portal circulation from the gut during portal triad occlusion. *Digestion*, **9**, 95–101.
6. Wang, X. D., Andersson, R., Soltesz, V., Bengmark, S. (1992) Bacterial translocation after major hepatectomy in patients and rats. *Arch. Surg.*, **127**, 1101–1106.

Bengt Jeppsson, MD, PhD
Associate Professor of Surgery
Department of Surgery
Lund University Hospital
S-221 85 LUND
SWEDEN
FAX: 46-46-13 97 76

### IS A CONSERVATIVE APPROACH JUSTIFIED IN PENETRATING LIVER INJURY?

### ABSTRACT

*Kundson, M.M., Lim, R.C. and Olcott, E.W. (1994) Morbidity and mortality following major penetrating liver injuries. Arch Surg; 129, 256–261.*

***Objective.*** **To establish the mortality and morbidity associated with major penetrating liver injuries and to describe the nature and treatment of complications related to these injuries. We postulated that there had been a trend toward less radical initial surgery, as well as an increased utilization of modern imaging techniques in both diagnosing and treating postoperative complications following penetrating liver trauma.**

***Design.*** **A retrospective survey of medical records and radiology files.**

***Setting.*** **A university trauma center in an urban setting.**

***Patients.*** **Of the 188 patients admitted to our trauma center with penetrating liver trauma between April 1988 and December 1991, 36 had major liver trauma (grades 3 through 5) and are described in this report.**

***Main Outcome Measures.*** **The mortality rate, type of operative treatment, and the nature and treatment of complications for each grade of major liver injury.**

***Results.*** **The mortality rate from major liver injuries was 17%. Surgical techniques employed primarily consisted of the use of hemostatic agents and cautery, simple suturing, direct vessel ligation, and packing. Fifty-two percent of the survivors had major complications related to the liver injury itself, but only two required operative therapy. The remaining patients were successfully treated with interventional radiologic techniques.**

***Conclusions.*** **The morbidity and mortality following major penetrating liver injuries remain significant. The majority of hepatobiliary complications can be successfully managed without further surgery bu t require the combined efforts of the surgeon and interventional radiologist. (Arch Surg. 1994, 129: 256-261)**

KEY WORDS: Liver trauma penetrating liver injury liver packing.

### PAPER DISCUSSION

In 1976 Lim and his colleagues, published what subsequently has come to be considered, a landmark in the field of hepatic trauma entitled - " Prevention of complications after liver trauma[1]." In that series, 129 patients sustaining both blunt and penetrating trauma comprised the cohort of patients analyzed. The authors documented that 17 of these patients (13%) were noted to have postoperative liver related complications, which necessitated reoperation in 75%. With an almost exponential rise in the technological advances in the field of radiologic imaging modalities (CAT scan, HIDA scan, Sonography, MRI) as well as the expertise and success exhibited by the "Interventional Radiologist" Knudson postulated that the necessity of operative intervention to solve postoperative complications would wane. This assumption, has proven to be correct. In a three and a half year period, which started in 1988 and ended in 1991, Knudson and her associates have accumulated 188 patients sustaining penetrating trauma at the San Francisco General Hospital. Of these, 36 patients (20%) sustained major hepatic injuries (Grades III-V)[2] and provide the subject matter of this report.

While the incidence of hepatic related complications in surviving patients has risen from 13% to 52% (n = 11), the incidence of reoperative surgery to correct these adverse sequelae has dramatically dropped to 18% ( n = 2). The almost five fold increase in the morbidity following surgical intervention to correct penetrating injuries to the liver reported by the authors is alarming. One might speculate that the overwhelming rise in availability of high powered weapons by teens and sub-teens and their almost indiscriminate use have been in some way responsible.

Knudson and her colleagues rightly emphasize the role of advanced imaging techniques and interventional radiology in limiting the need for surgical solutions to post operative complications resulting from the operative management of complex penetrating hepatic injuries. Nevertheless, some additional confirmatory thoughts on this matter may be of use to the reader.

The combination of high speed computerized scanners in diagnosing postoperative intraabdominal abscesses (accuracy rates approaching 100%)[3,4] as well as the therapeutic achievements attained by interventional radiologists has without question made an extraordinary impact on the way major traumatic hepatic injuries are managed in 1994. Percutaneous aspiration and drainage of unilocular abscesses has been successful in 95% of reported instances-leading to the avoidance of what surgeons a decade ago dreaded, but were nevertheless compelled by necessity to perform, that of reexploring a complicated trauma patient in the early postoperative period. Equally important is the ability of the interventional radiologist to drain " bilomas", and stent lacerated bile ducts, as a temporizing maneuver, in critically ill patients obviating the need for immediate surgical intervention. Often the stenting procedure in of itself can be curative as the injured duct heals over the percutaneous catheter. Should stricture formation of the bile duct complicate the healing process, therapeutic results have been achieved via percutaneous balloon dilatation or the placement into these narrowed bile ducts of self expanding stainless steel endoprostheses[5,6].

No less significant in the management of complications arising after complex hepatic injuries have occurred are the advances made in the field of angiography. Pseudoaneursyms of the main hepatic arteries or their branches, with the subsequent development of hematobilia is an uncommon, but well recognized complication following traumatic injuries to the liver[7]. Angiography has been the mainstay in making the diagnosis of this elusive syndrome. In the past, once the diagnosis was made, operative intervention was the only therapeutic modality available to deal with this complication, often resulting in either a lobar or formal anatomic hepatic resection. Over the past 15 years, however, therapeutic angio-embolization of the offending vessel with steel coils, Gelfoam or acrylate glue has emerged as the preferred treatment once the diagnosis of hemobilia has been confirmed[8]. With experience, angio-embolization has been extended to the treatment of traumatic arterio-portal venous fistulas[9]. Left untreated this entity will lead to gastrointestinal bleeding, initially as a result of mucosal edema and subsequently from variceal bleeding as collaterals develop with ensuing portal hypertension[9].

Aside from the major thesis presented in this paper some additional relevant gems can be garnered from the manuscript. For instance, the authors noted that all nine patients undergoing emergency room thoracotomy expired. This sobering detail brings into question, once again, both the role and efficacy of resorting to an ER thoracotomy for patients sustaining penetrating abdominal injuries. Additionally, the authors reaffirm that penetrating complex hepatic injuries continue, even in 1994, to carry a high mortality rate, 42% reported in this series, and that juxta-hepatic vascular injuries are almost always lethal. On a more practical level the authors observed that patients with complex liver injuries (Grades III-V) were particularly prone to the development of postoperative biliary tract complications and thus should routinely be managed by closed suction drainage. This pronouncement was made in full cognizance of the controversy surrounding the role of drains in hepatic trauma. Recent reports examining routine drainage in the management of hepatic injuries concluded that : 1) they were unnecessary; and 2) significantly increased the likelihood of septic complications [3,4,10]. Closer analysis of the available data, however, indicates that "open drainage" (Penrose or sump) is associated with the highest incidence of postoperative abscess formation. Closed suction drainage (Jackson-Pratt), on the other hand, is less likely to result in abscess formation than either open suction or no drainage at all[3,10,11]. Injuries classified as Grades I-II can be managed without drainage provided that hemostasis has been achieved and obvious bile leaks are not apparent. Grade III injuries or above, as the author rightly assert, should be drained. Support for this conclusion is based on data accrued from the multicenter study by Cogbill[12] as well as the results achieved at the Bellevue Trauma and Shock Unit[13]. Objections to the contrary, a combined postoperative abscess rate in the two aforementioned series of 8.2%, when drainage was employed in 97% of all grade III injuries or above is difficult to argue with.

An additional issue alluded to, but not elaborated upon, in the manuscript is the critical role played by the pivotal maneuver of packing and planned reexploration. This maneuver becomes lifesaving when confronted with complex hepatic injuries refractory to the more traditional methods of treatment and complicated by an ongoing coagulopathy. There also seems to be a census among Level I trauma surgeons that packing and planned reexploration, usually within 36 hours, is necessary in only 3–5% of all complex hepatic injuries as more conventional methods of controlling hemorrhages would have sufficed. The three patients in this series where perihepatic packing was employed undoubtedly would have succumbed had the surgeons not resorted to this technique.

Lastly, the editors have posed, in an oblique fashion, the rather provocative question whether a conservative approach is justified in penetrating liver injuries? Presumably, their question was prompted by the two CAT scans provided by Knudson depicting remarkable healing of two major hepatic injuries resulting from gunshot wounds. Several factors should, however, be noted: 1) The authors by no means advocate non-operative management of penetrating injuries: 2) both of the original scans were obtained in the postoperative period, and data regarding intraoperative management as well as indications for postoperative scanning in either of these patients is lacking. Previous studies have indicated that if the mechanism of injury is a missile penetrating the peritoneal cavity rather than a knife, damage to a visceral or a vascular structure is apt to have occurred in 96–98% [14]. Under these circumstances, surgical intervention is warranted even in the presence of minimal physical findings as the odds that the patient sustained a significant injury are overwhelming. Excellent results attained with the non operative management of blunt hepatic injuries notwithstanding, extolling the ability of the liver to undergo spontaneous healing even in face of major liver trauma is fraught with danger when extrapolated to penetrating hepatic injuries and should not, at present, be advocated. A conservative approach, however, in the management of postoperative complications resulting from penetrating hepatic injuries, as Knudson has so nicely demonstrated, is justified. Intuitively Level I trauma centers have felt this to be the case, but it is always comforting to have the supporting data, even though purists may find fault with conclusions based on retrospective analysis.

*References*

1. Lim, R.C., Lau G., Steele, M. (1976) Prevention of complication after liver trauma. *Am. J. Surg.*, **132**, 156–162.
2. Moore, E.E., Shackford, S.R., Pachter, H.L. *et al.* (1989) Organ injury scaling: spleen liver and kidney. *J. Trauma.*, **29**, 1664–1666.
3. Fabian, T. C., Croce, M.A., Stanford, G.C., *et al.* (1991) Factors affecting morbidity following hepatic trauma: a prospective analysis of 482 liver injuries. *Ann. Surg.*, **213**, 540–548.
4. Bender, J. S., Geller, E.R., Wilson, R. F. (1989) Intra-abdominal sepsis following liver trauma. *J. Trauma.*, **29**, 1140–1145
5. Dick, R., Gilliams, A., Dooley, J.S., Hobbs, K.E.F. (1989) Stainless steel mesh stents for biliary stricture. J. *Intervent. Rad.*, **4**, 95–98.
6. Gilliams, A., Dick, R., Dooley, J.S., *et al.* (1990) Self expandable stainless steel brainded endoprosthesis for biliary stricture. *Rad.*, **174**, 137–140.
7. Sandbloom, R., Saegesser, F., Mirkovitch, V. (1984) Hepatic hemobilia: Hemorrhage from the intrahepatic biliary tract, A review World. *J. Surg.*, **8**, 41–50.
8. Cyret, P., Baumer, R., Roche, A. (1984) Hepatic hemobilia of traumatic or iatrogenic origin. Recent advances of diagnosis and therapy. Review of the literature from 1976–1981. *World J. Surg.*, **8**, 2.
9. Tanaka, H., Iwai, A., Sugimoto, H. (1991) Intrahepatic arterioportal fistula after blunt hepatic trauma: case reports. *J. Trauma.*, **31**, 143–146.
10. Noyes, L.D., Doyle, D.J., McSwain, N.E. (1988) Septic complications associated with the use of peritoneal drains in liver traumas. *J. Trauma.*, **28**, 337.
11. Gillmore, D., McSwain, N. E. Jr., Browder, I.W., (1987) Hepatic trauma: To drain or not to drain? *J. Trauma.*, **27**, 898.
12. Cogbill, T.H., Moore, E.E., Jurkovitch, G.J. *et al.* (1988) A multi-center experience with 1, 335 liver injuries. *J. Trauma.*, **28**, 14–33.
13. Pachter, H.L., Spencer, F.C., Hofstetter, S.R., Liang, H.G., Coppa, G.F. (1992) Significant trends in the treatment of hepatic trauma: Experience with 411 injuries. *Ann. Surg.*, **215**, 492–502.
14. Moore, E.E., Moore, J.B., Van Duzzer- Moore, S. *et al.* (1980) Mandatory laparotomy for gunshot wounds penetrating the abdomen. *Am. J. Surg.*, **140**, 847.

H. Leon Pachter, M D
Professor of Surgery
New York University School Of Medicine
Director, Trauma-Shock Unit
Bellevue Hospital, New York City, N.Y.

### NARROW VERSUS WIDE DIAMETER PORTACAVAL H-GRAFT SHUNTS

### ABSTRACT

*Sarfeh James, I. and Rypins, Eric, B. (1994) Partial versus total portacaval shunt in alcoholic cirrhosis. Annals of Surgery; 219, 353–361.*

***Objective.*** **Results of the first prospective randomized clinical trial comparing partial and total portacaval shunt for variceal hemorrhage are reported.**
***Summary Background Data.*** **Total portacaval shunts produce subnormal portal pressures, completely diverting hepatic portal flow. Partial shunts maintain higher pressures and preserve hepatopedal flow. No randomized trials of these two approaches have been performed.**
***Methods.*** **Alcoholic patients with cirrhosis (n = 30) and variceal hemorrhage treated at one institution were randomized to receive partial (8-mm diameter portacaval H grafts with collateral ablation, n = 14) or total shunts (16-mm diameter grafts, n = 16). Portography was performed after operation and then yearly. Investigators blinded to shunt type assessed encephalopathy; hospitalizations were reviewed.**
***Results.*** **Child's class, age and operative urgency were similar for the two groups. Two patients (with total shunts) died within 30 days. Hepatopedal flow was maintained in 13 partial and 0 total shunt patients ($p < 0.0001$). Shunt gradients were 16 ± 5 compared with 6 ± 3 cm saline after partial and total shunts ($p < 0.0001$). There were no shunt thromboses or variceal hemorrhages. Encephalopathy-free survival was significantly greater after partial shunts ($p = 0.013$; life table analysis). Five total compared with zero partial shunt patients required hospitalization for coma ($p = 0.02$). Long-term survival was not different for the two groups of patients.**
***Conclusions.*** **Partial shunts control variceal hemorrhages while maintaining hepatopedal flow and elevated portal pressures. By minimizing encephalopathy rates, partial shunts provide improved quality of survival compared with total shunts.**

KEY WORDS: Portacaval shunt sarfeh shunt.

### PAPER DISCUSSION

Partial portal decompression - does it really work? This carefully conducted prospective randomized controlled trial comparing partial to total portal decompression presents the most persuasive data yet available that partial decompression offers a significant advantage compared to total portal systemic shunt. This study needed to be done, and the authors are to be commended for a well designed and carefully executed study[1]. As two of the major

